# Hsa_circRNA_0000518 facilitates breast cancer development via regulation of the miR‐326/FGFR1 axis

**DOI:** 10.1111/1759-7714.13641

**Published:** 2020-10-01

**Authors:** Jing Jiang, Hui Lin, Shenghong Shi, Ying Hong, Xianan Bai, Xuchen Cao

**Affiliations:** ^1^ The First Department of Breast Cancer Tianjin Medical University Cancer Institute Hospital, National Clinical Research Center for Cancer Tianjin China; ^2^ Key Laboratory of Cancer Prevention and Therapy Tianjin China; ^3^ Tianjin's Clinical Research Center for Cancer Tianjin China; ^4^ Key Laboratory of Breast Cancer Prevention and Therapy Tianjin Medical University, Ministry of Education Tianjin China; ^5^ Department of Breast Surgery, HwaMei Hospital University of Chinese Academy of Sciences Ningbo China; ^6^ Ningbo Institute of Life and Health Industry University of Chinese Academy of Sciences Ningbo China; ^7^ Department of Thyroid and Breast Surgery Taizhou Hospital of Zhejiang Province Affiliated to WenZhou Medical University Taizhou China

**Keywords:** Breast cancer, circ_0000518, FGFR1, miR‐326

## Abstract

**Background:**

Breast cancer (BC) is a heterogeneous malignant tumor that threatens the health of women worldwide. Hsa_circRNA_0000518 (circ_0000518) has been revealed to be upregulated in BC tissues. However, the role and mechanism of circ_0000518 in BC are indistinct.

**Methods:**

Quantitative real‐time polymerase chain reaction (qRT‐PCR) was implemented to detect the levels of circ_0000518, microRNA (miR)‐326, and fibroblast growth factor receptor 1 (FGFR1) mRNA in BC tissues and cells. Cell counting kit‐8 (CCK‐8), colony formation, flow cytometry, and transwell assays were executed to estimate BC cell proliferation, cell cycle progression, apoptosis, migration, and invasion. The relationship between circ_0000518 or FGFR1 and miR‐326 was verified by dual‐luciferase reporter and/or RNA immunoprecipitation (RIP) assays. The role of circ_0000518 in vivo was confirmed by xenograft assay.

**Results:**

Circ_0000518 and FGFR1 were upregulated while miR‐326 was downregulated in BC tissues and cells. Circ_0000518 silencing impeded tumor growth in vivo and induced cell cycle arrest, apoptosis, cured proliferation, colony formation, migration, and invasion of BC cells in vitro. Circ_0000518 regulated FGFR1 expression via competitively binding to miR‐326 in BC cells. MiR‐326 inhibitor reversed the inhibitory influence of circ_0000518 knockdown on the malignant behaviors of BC cells. FGFR1 overexpression abolished miR‐326 mimic‐mediated influence on the malignant behaviors of BC cells.

**Conclusions:**

Circ_0000518 facilitated BC development via regulation of the miR‐326/FGFR1 axis, suggesting that circ_0000518 might be a promising target for BC treatment.

## Graphical abstract

The research exhibited the critical role of circ_0000518 in BC. Circ_0000518 accelerated BC progression via elevating FGFR1 expression by sponging miR‐326. The findings highlighted the prospect of circ_0000518 as a new target for BC treatment.

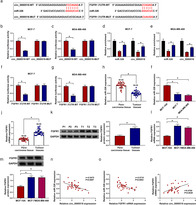

## Introduction

Breast cancer (BC) is a heterogeneous malignant tumor in women worldwide.^1^ In 2018, 2.1 million new cases of BC were diagnosed worldwide, accounting for about a quarter of female cancer cases.^2^ At present, primary or neoadjuvant chemotherapy combined with local therapy (surgery or radiotherapy) and postoperative systemic chemotherapy have become accepted strategies for BC treatment.^3^ Because of the high frequency of drug resistance, metastasis, and relapse, the prognosis of BC patients has not been overtly improved.^4^, ^5^ Therefore, it is necessary to further our understanding of the molecular mechanism of BC to formulate more effective treatment strategies.

Circular RNAs (circRNAs), a type of non‐coding RNA with a covalently closed circular structure, exert vital biological functions through serving as protein decoys or microRNA (miR) sponges.^6^ CircRNA is more stable than its linear counterpart because it can resist RNase R degradation.^7^ CircRNAs have been demonstrated to exert a vital role in the development of diverse diseases.^8^, ^9^ For example, circRNA circ_SMC3 has been reported to accelerate the tumorigenesis of gastric cancer.^10^ We analyzed high‐throughput circRNA microarray data (GSE101123 database) and found that circ_0000518 was a differentially expressed circRNA in BC. Hsa_circRNA_0000518 (circ_0000518) is formed by the reverse splicing of the ribonuclease P RNA component H1 (RPPH1) gene, and its length is 150 bp. At present, the role of circ_0000518 in BC is unknown.

MiRs are short non‐coding RNAs (about 19–25 nucleotides) that exert an important role in cellular processes for normal development.^11^ Increasing evidence has indicated that miRs are related to the advancement of various tumors.^12^ MiR‐326 has been revealed to take part in embryonic development, oncogenesis, chemotherapy resistance, immunomodulation, and cell invasion.^13^ MiR‐326 has also been identified as a tumor suppressor in a range of tumors, such as papillary thyroid cancer^14^ and oral squamous cell cancer,^15^ as well as being associated with BC development. However, the mechanism by which miR‐326 modulates the advancement of BC has so far not been clearly explained.

Fibroblast growth factor receptor 1 (FGFR1) is a member of the FGFR family, which activates PI3K/AKT signaling and MAPK signaling via acting as a receptor for tyrosine kinase.^16^ In gastric cancer, FGFR1 overexpression has been found to contribute to peritoneal dissemination.^17^ Moreover, FGFR1 has been demonstrated to facilitate lung cancer cell metastasis and epithelial‐mesenchymal transition.^18^ FGFR1 signaling has been reported as a vital pathway to drive BC metastasis to the lungs.^19^ However, the mechanism of FGFR1 signaling involved in BC progression has not been fully elucidated.

Herein, we discovered that circ_0000518 plays a carcinogenic role in BC, and moreover, was able to facilitate BC progression via regulation of the miR‐326/FGFR1 axis.

## Methods

### Patient‐derived samples

Clinical BC tissue samples were collected, including BC tissues and paracarcinoma tissues, from BC patients who accepted surgery in Tianjin Medical University Cancer Institute Hospital. The BC patients who underwent radiotherapy or chemotherapy were excluded in terms of the inclusion criteria. Written informed consents were provided by all enrolled patients. The research was ratified by the Ethics Committee of Tianjin Medical University Cancer Institute Hospital.

### Cell culture and transfection

Human normal breast cell line MCF‐10A and BC cell lines MCF‐7 and MDA‐MB‐468 were acquired from American Type Culture Collection (Manassas, VA, USA). MCF‐10A cells were cultured in MEBM BulletKit (Lonza, Basel, Switzerland). MCF‐7 and MDA‐MB‐468 cells were cultured in Dulbecco's modified Eagle's medium (DMEM, Invitrogen, Sigma, Louis, Missouri, MO, USA) supplemented with fetal bovine serum (FBS, 10%, Sigma), streptomycin (100 μg/mL, Sigma), and penicillin (100 U/mL, Sigma). These cells were kept in a moist atmosphere with 5% CO_2_ at 37°C.

Small interference (si) RNA targeting circ_0000518 (si‐circ_0000518) and corresponding control (si‐NC), as well as miR‐326 mimic and inhibitor (miR‐326 and anti‐miR‐326) and their matching negative controls (miR‐NC and anti‐miR‐NC), were obtained from GenePharma (Shanghai, China). The overexpression vectors of FGFR1 (pcDNA‐FGFR1) and circ_0000518 (circ_0000518) were established via cloning the full‐length sequence of FGFR1 or circ_0000518 into the pcDNA3.1 vector (pcDNA‐NC) (Invitrogen, Carlsbad, CA, USA) or pcD‐ciR (Geneseed Biotech Co., Ltd., Guangzhou, China) (Vector). BC cells (MCF‐7 and MDA‐MB‐468) were transfected with oligonucleotides or vectors using Lipofectamine 3000 reagent (Life Technologies, Grand Island, NY, USA).

### Quantitative real‐time polymerase chain reaction (qRT‐PCR)

Total RNA was extracted from tissue samples and cells with RNAiso Plus (Takara, Dalian, China). Next, the complementary DNA was synthesized with the PrimeScript RT reagent Master Mix (Takara) or Mir‐X miRNA First‐Strand Synthesis Kit (Takara). QRT‐PCR was executed by using a Light Cycler 480 II Real‐Time PCR System (Roche, Basel, Switzerland) with the SYBR Premix Ex Taq kit (Takara). The 2^−ΔΔCt^ method was utilized to calculate the expression of circ_0000518, miR‐326, and FGFR1, and glyceraldehyde‐3‐phosphate dehydrogenase (GAPDH) or U6 small nuclear RNA (snRNA) was used as the control. The primers were used as below: circ_0000518: (F:5′‐AGGTGAGTTCCCAGAGAACGG‐3′ and R:5′‐AGTGGAGTGACAGGACGCA‐3′); GAPDH: (F:5′‐GACTCCACTCACGGCAAATTCA‐3′ and R:5′‐TCGCTCCTGGAAGATGGTGAT‐3′); miR‐326: (F:5′‐CCTCTGGGCCCTTCCTCCAG‐3′ and R:5′‐GCTGTCAACGATACGCTACCTA‐3′); U6 snRNA (F:5′‐GCTCGCTTCGGCAGCACA‐3′ and R:5′‐GAGGTATTCGCACCAGAGGA‐3′); FGFR1: (F:5′‐CCCGTAGCTCCATATTGGACA‐3′ and R:5′‐TTTGCCATTTTTCAACCAGCG ‐3′).

### Cell proliferation assay

After transfection with oligonucleotides or vectors, the BC cells (5 × 10^3^ cells/well) were cultured for 24, 48, or 72 hours under the right conditions. Next, 10 μL cell counting kit‐8 (CCK‐8) reagent (Dojindo, Tokyo, Japan) was complemented into each well following the manufacturer's protocol. Thereafter, the absorbance at 450 nm was measured with the microplate absorbance reader (Bio‐Rad, Hercules, CA, USA).

### Cell colony formation assay

The transfected BC cells (5 × 10^3^) were seeded in a six‐well plate with DMEM and cultured for 14 days. Subsequently, the cells were fixed with paraformaldehyde (4%) and stained with crystal violet (0.5%, Beyotime, Shanghai, China). After washing with phosphate buffer solution (PBS), the colonies (>50 cells) were counted and photographed via light microscope (Olympus, Tokyo, Japan).

### Cell cycle and apoptosis analysis

For cell cycle assay, the BC cells were transfected with appointed oligonucleotides or vectors. After culture for 48 hours, the cells were treated with propidium iodide (PI) (50 μg/mL, Sigma) solution containing sodium citrate (0.1%) and Triton X‐100 (0.1%). For cell apoptosis assay, the transfected BC cells were treated with the FITC‐Annexin V Apoptosis Detection Kit (Dojindo). Cell cycle and apoptosis were estimated with a FACScalibur flow cytometer (BD Biosciences, San Jose, CA, USA).

### Cell migration and invasion analysis

The migration capacity of the transfected BC cells was evaluated with an 8 μm pore membrane filter (Costar, Cambridge, MA, USA). In brief, the DMEM (serum‐free) containing BC cells (1 × 10^5^ cells) was added to the upper chamber. The lower chamber was supplemented with the DMEM containing 10% FBS. After incubation for 24 hours, the cells on the lower surface of the membrane were fixed with paraformaldehyde (4%) and stained with crystal violet (0.5%, Beyotime). Thereafter, the cells were visualized and counted with a light microscope (Olympus) at 100 × magnification. The cell invasion test was performed in the same way as the migration test, except that the 8 μm pore membrane filter used in the cell invasion test was precoated with Matrigel (BD Biosciences).

### Dual‐luciferase reporter assay

The binding sites between circ_0000518 or FGFR1 and miR‐326 were predicted by using the circInteractome or targetscan databases. The sequences of wild‐type circ_0000518 (circ_0000518‐WT), mutant circ_0000518 (circ_0000518‐MUT), wild‐type 3′ untranslated regions (UTR) of FGFR1 (FGFR1 3′UTR‐WT), and mutant 3′UTR of FGFR1 (FGFR1 3′UTR‐MUT) were inserted into the pmirGLO luciferase vectors (GeneCreat, Wuhan, China) to construct the luciferase reporters, respectively. Then, the luciferase reporters were cotransfected into BC cells with miR‐NC or miR‐326. The luciferase intensities were determined with the luciferase reporter assay kit (Promega, Madison, WI, USA) by normalizing the firefly luminescence to Renilla luminescence.

### RNA immunoprecipitation (RIP) assay

The specific binding circ_0000518 and miR‐326 were verified with a Magna RIP kit (Millipore, Bedford, MA, USA). In short, BC cells were lysed in complete RNA lysis buffer. Thereafter, the lysate was incubated with RIP immunoprecipitation buffer including protein A/G sepharose beads conjugated with IgG antibody (ab109489, 1:100, Abcam, Cambridge, MA, USA) or Ago‐2 antibody (ab186733, 1:50, Abcam). The immunoprecipitated RNA was extracted using the RNeasy Mini Kit (Qiagen). QRT‐PCR was utilized to analyze the enrichment of circ_0000518 and miR‐326 in the immunoprecipitated RNA.

### Western blotting

Total protein was extracted from tissue samples and cells with RIPA lysis buffer containing protease inhibitor (Beyotime). Total protein was separated by using sodium dodecyl sulfate‐polyacrylamide gel electrophoresis (SDS‐PAGE). Thereafter, the separated proteins were transferred to polyvinylidene difluoride (PVDF) membranes (Beyotime), and the membranes were blocked with tris buffered saline tween buffer with 5% skim milk. The membranes were then incubated with primary antibodies, including anti‐FGFR1 (ab76464, 1:500, Abcam) and anti‐GAPDH (ab9484, 1:1000, Abcam). GAPDH was deemed as a loading control. Next, the membranes were incubated with the secondary antibody (ab6721, 1:5000, Abcam). The immunoblots were visualized with enhanced chemiluminescence solution (Beyotime).

### Xenograft assay

Circ_0000518 short hairpin RNA (sh‐circ_0000518) and matching control (sh‐NC) were purchased from GenePharma. The circ_0000518‐knockdown MCF‐7 cells were constructed through infecting with the hU6‐MCS‐CMV‐Puromycin lentiviral vectors with sh‐circ_0000518. The MCF‐7 cells (1 × 10^7^ cells/0.2 mL PBS) carrying sh‐circ_0000518 or sh‐NC were subcutaneously injected into the right flank of 10 BALB/c nude mice (5‐week‐old, Experimental Animal Center, Shanghai, China) (five mice/group). All nude mice were fed under specific pathogen free conditions. The mice were sacrificed by cervical dislocation under 5% isoflurane to acquire their tumor tissues after injection after 27 days. Tumor volume was measured every four days using a caliper. Tumor volume was calculated in light of the following equation: Volume = (length × width^2^)/2. The protocols of tumor formation experiments were approved by the Animal Ethics Committee of Tianjin Medical University Cancer Institute Hospital.

### Statistical analysis

All in vitro experiments in this study were repeated at least three times. Statistical analysis was implemented with GraphPad Prism 6 software (GraphPad, San Diego, CA, USA). Data are shown as mean ± standard deviation. The normal distribution of the data was determined with a Kolmogorov‐Smirnov test. Differences were deemed significant if *P* < 0.05. The differences between two groups were determined with Student's *t*‐test, and three or more groups were estimated by using one‐way variance analysis (ANOVA) with hoc post Turkey test. The homogeneity of the variances was analyzed with the F‐test.

## Results

### Microarray data of circRNAs differentially expressed in BC

**Figure 1:**
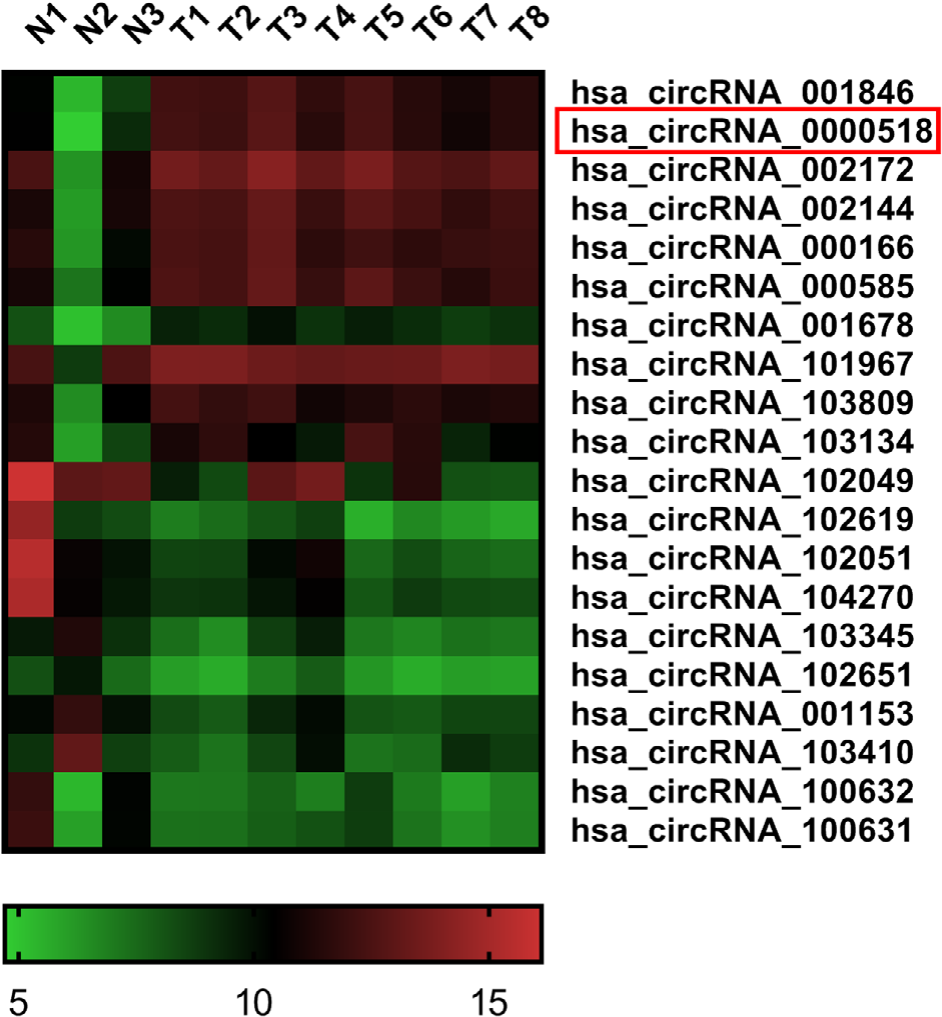
Microarray data of 20 differentially expressed circRNAs in BC. Heat map exhibiting 20 differentially expressed circRNAs (GSE101123 database).

To identify differentially expressed circRNAs in BC, we analyzed high‐throughput circRNA microarray data (GSE101123 database). We selected 20 circRNAs that were significantly upregulated or downregulated in BC tissues and then plotted a heat map (Fig 1). Among 20 differentially expressed circRNAs, we selected five circRNAs (hsa_circRNA_001846, hsa_circRNA_000518, hsa_circRNA_002172, hsa_circRNA_002144, and hsa_circRNA_000166) for preliminary experiment. QRT‐PCR showed that hsa_circRNA_001846, hsa_circRNA_000518, and hsa_circRNA_000166 were significantly upregulated in BC tissue samples (10 random samples). We selected hsa_circRNA_000518, which had a relatively larger expression change, as the research object (Fig S1).

### Circ_0000518 accelerated the malignancy of BC cells

**Figure 2:**
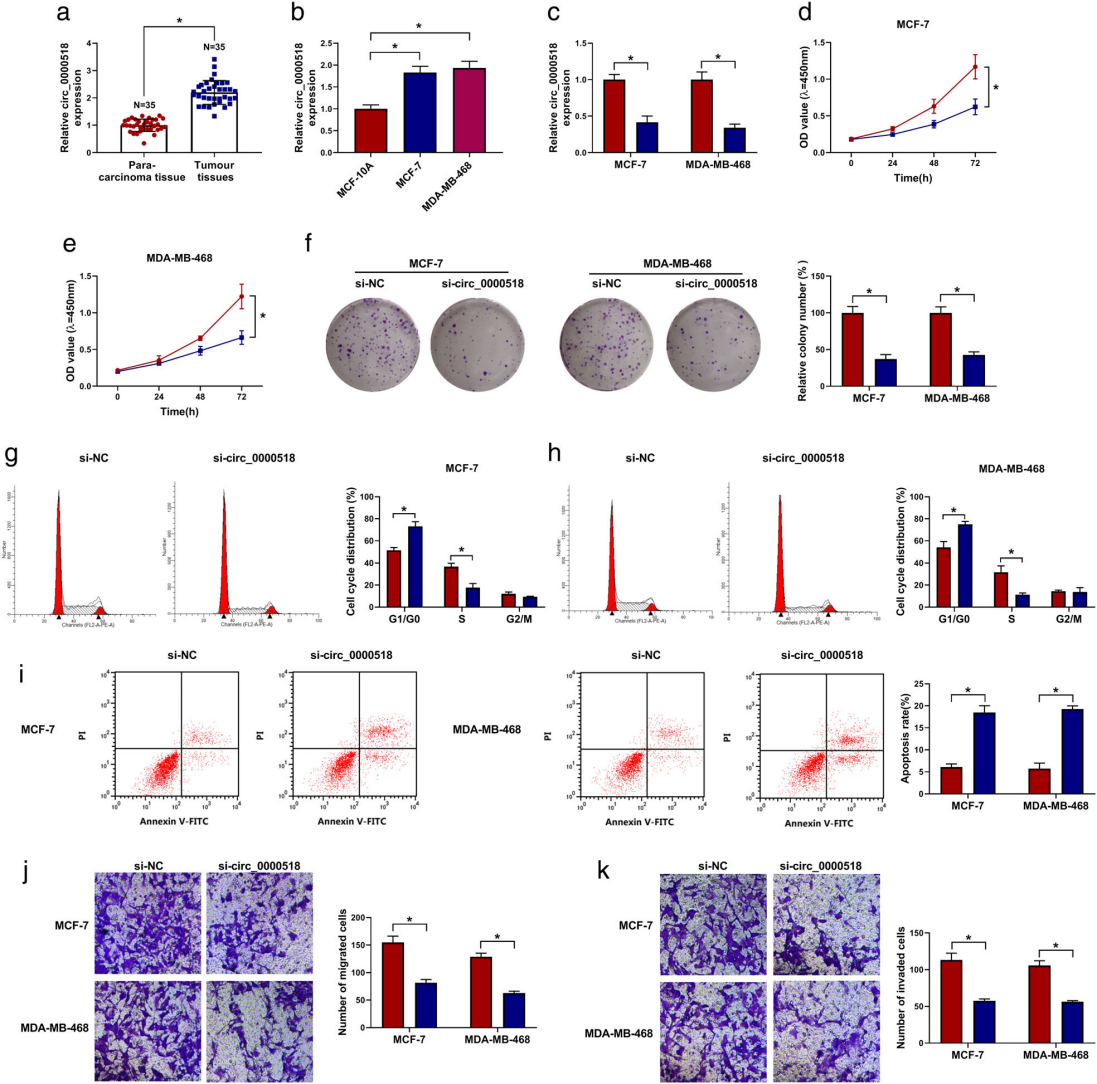
Effects of circ_0000518 knockdown on cell proliferation, colony formation, cell cycle progression, apoptosis, migration, and invasion in BC cells. (**a** and **b**) QRT‐PCR was performed to examine the expression of circ_0000518 in 35 paired BC tissues and para‐carcinoma tissues, as well as BC cell lines (MCF‐7 and MDA‐MB‐468) and MCF‐10A cell line. (**c**–**k**) The proliferation, colony formation, cell cycle progression, apoptosis, migration, and invasion of MCF‐7 and MDA‐MB‐468 cells after si‐circ_0000518 or si‐NC transfection were determined with CCK‐8, colony formation, flow cytometry, or transwell assays. The experiments were repeated three times. Data are shown as mean ± standard deviation. *P < 0.05. (**c**) (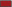) si‐NC and (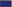) si‐circ_0000518. (**d**) MCF‐7 (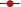) si‐NC and (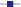) si‐circ_0000518. (**e**) MDA‐MB‐468 (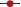) si‐NC and (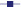) si‐circ_0000518. (**f**) (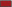) si‐NC and (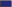) si‐circ_0000518. (**g**) si‐NC (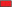) si‐NC, (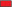) si‐NC, and (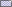) si‐circ_0000518 and si‐circ_0000518 (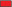) si‐NC, (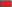) si‐NC, and (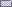) si‐circ_0000518. MCF‐7 (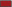) si‐NC and (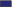) si‐circ_0000518. (**h**) si‐NC (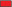) si‐NC, (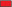) si‐NC, and (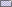) si‐circ_0000518 and si‐circ_0000518 (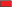) si‐NC, (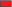) si‐NC, and (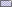) si‐circ_0000518. MDA‐MB‐468 (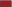) si‐NC and (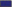) si‐circ_0000518. (**i**) (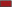) si‐NC and (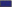) si‐circ_0000518. (**j**) (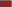) si‐NC and (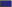) si‐circ_0000518. (**k**) (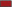) si‐NC and (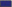) si‐circ_0000518.

To explore the biological function of circ_0000518 in BC, we examined the levels of circ_0000518 in 35 paired BC tissues and para‐carcinoma tissues. QRT‐PCR manifested that circ_0000518 expression was increased in BC tissues in contrast to the paracarcinoma tissues (Fig 2a). Circ_0000518 expression was also elevated in BC cell lines (MCF‐7 and MDA‐MB‐468) relative to the MCF‐10A cell line (Fig 2b). The knockdown efficiency of circ_0000518 is shown in Fig 2c. Subsequently, CCK‐8 assay indicated that circ_0000518 downregulation could curb the proliferation of MCF‐7 and MDA‐MB‐468 cells (Fig 2d,e). Cell colony formation assay manifested that the colony number of MCF‐7 and MDA‐MB‐468 cells was decreased by circ_0000518 inhibition (Fig 2f). Flow cytometry assay showed that silenced circ_0000518 expression arrested cell cycle progression and induced cell apoptosis in MCF‐7 and MDA‐MB‐468 cells (Fig 2g–i). Transwell assay revealed that circ_0000518 silencing repressed cell migration and invasion in MCF‐7 and MDA‐MB‐468 cells (Fig 2j,k). We also explored the influence of circ_0000518 overexpression on the malignancy of BC cells. The overexpression efficiency of circ_0000518 is shown in Fig S2A. Also, the overexpression of circ_0000518 reduced cell apoptosis and accelerated cell proliferation, colony formation, cell cycle progression, migration, and invasion in BC cells (Fig S2B–I). These finding manifested that circ_0000518 facilitated the malignant behavior of BC cells.

### Circ_0000518 identified as a sponge for miR‐326, which targeted FGFR1 in BC cells

**Figure 3:**
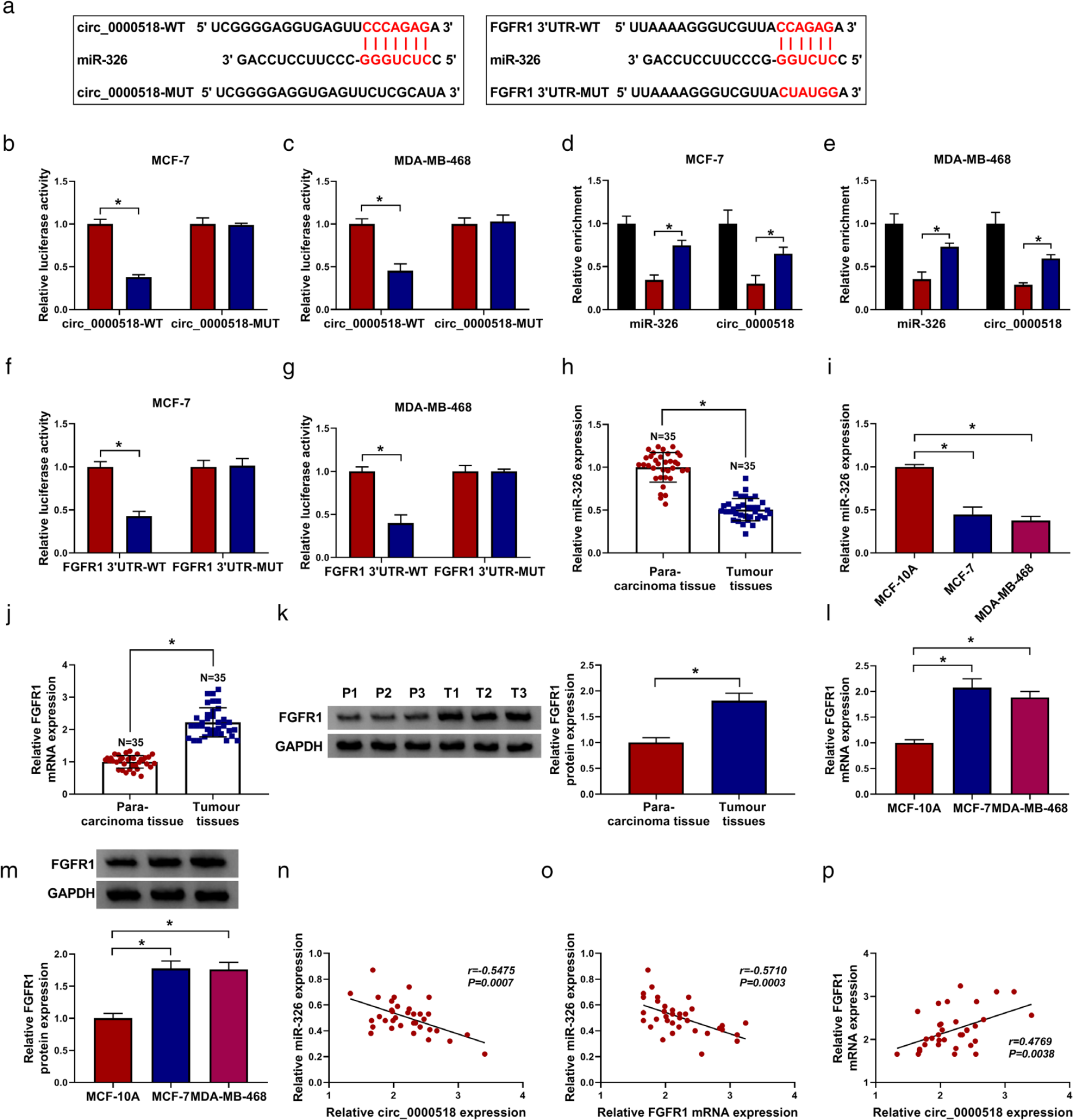
Circ_0000518 acted as a sponge for miR‐326, which targeted FGFR1 in BC cells. (**a**) The binding sites between miR‐326 and circ_0000518 or FGFR1 were predicted with circInteractome or targetscan databases. (**b** and **c**) Dual‐luciferase reporter assay was conducted to evaluate the luciferase activity of luciferase reporters with circ_0000518‐WT or circ_0000518‐MUT in MCF‐7 and MDA‐MB‐468 cells transfected with miR‐326 or miR‐NC. (**d** and **e**) After RIP assay, the enrichment of miR‐326 and circ_0000518 was examined using qRT‐PCR. (**f** and **g**) Dual‐luciferase reporter assay revealed the luciferase activities of the FGFR1 3′UTR‐WT and FGFR1 3′UTR‐MUT reporters in MCF‐7 and MDA‐MB‐468 cells transfected with miR‐326 or miR‐NC. (**h** and **i**) Expression levels of miR‐326 in BC tissues and para‐carcinoma tissues, as well as BC (MCF‐7 and MDA‐MB‐468) and MCF‐10A cells, were assessed by qRT‐PCR. (**j**–**m**) The mRNA and protein levels of FGFR1 in BC tissues and para‐carcinoma tissues, as well as BC (MCF‐7 and MDA‐MB‐468) and MCF‐10A cells, were detected by qRT‐PCR or western blotting. (**n**–**p**) The correlation among miR‐326, circ_0000518, and FGFR1 in BC tissues was determined via Pearson's correlation analysis. The experiments were repeated three times. Data are shown as mean ± standard deviation. *P < 0.05. (**b**) MCF‐7 (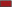) miR‐NC and (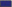) miR‐326. (**c**) MDA‐MB‐468 (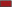) miR‐NC and (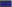) miR‐326. (**d**) MCF‐7 (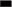) input, (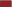) anti‐IgG, and (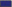) anti‐Ago2. (**e**) MDA‐MB‐468 (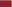) anti‐IgG, (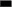) input, and (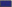) anti‐Ago2. (**f**) MCF‐7 (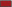) miR‐NC and (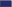) miR‐326. (**g**) MDA‐MB‐468 (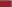) miR‐NC and (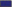) miR‐326.

Subsequently, we further explored the molecular mechanism of circ_0000518 in BC. We found that miR‐326 had the potential binding sites for circ_0000518 through using the circInteractome database. Targetscan database showed that miR‐326 had complementary sites to FGFR1 (Fig 3a). Dual‐luciferase reporter assay revealed that miR‐326 elevation inhibited the luciferase intensity of the luciferase reporters with circ_0000518‐WT in MCF‐7 and MDA‐MB‐468 cells compared to the control miR‐NC, while there was no overt change in the luciferase reporters with circ_0000518‐MUT (Fig 3b,c). RIP assay exhibited that circ_0000518 and miR‐326 were preferentially enriched in Ago2‐containing complexes compared to the control group (Fig 3d,e). Also, the luciferase activity of the FGFR1 3′UTR‐WT reporter was suppressed by miR‐326 mimic in MCF‐7 and MDA‐MB‐468 cells, but the luciferase activity of the FGFR1 3′UTR‐MUT reporter did not change (Fig 3f,g). Furthermore, miR‐326 expression was decreased in BC tissues and cell lines in comparison to their matching controls (Fig 3h,i). Inversely, FGFR1 mRNA and protein levels were elevated in BC tissues and cell lines (Fig 3j–m). The expression of miR‐326 and circ_0000518 or FGFR1 mRNA in BC tissues had a negative correlation (Fig 3n,o). However, the expression of FGFR1 mRNA was positively correlated with circ_0000518 in BC cells (Fig 3p). These results indicated that circ_0000518 served as a sponge for miR‐326, which targeted FGFR1 in BC cells.

### Circ_0000518 regulated FGFR1 expression through competitively binding to miR‐326 in BC cells

**Figure 4:**
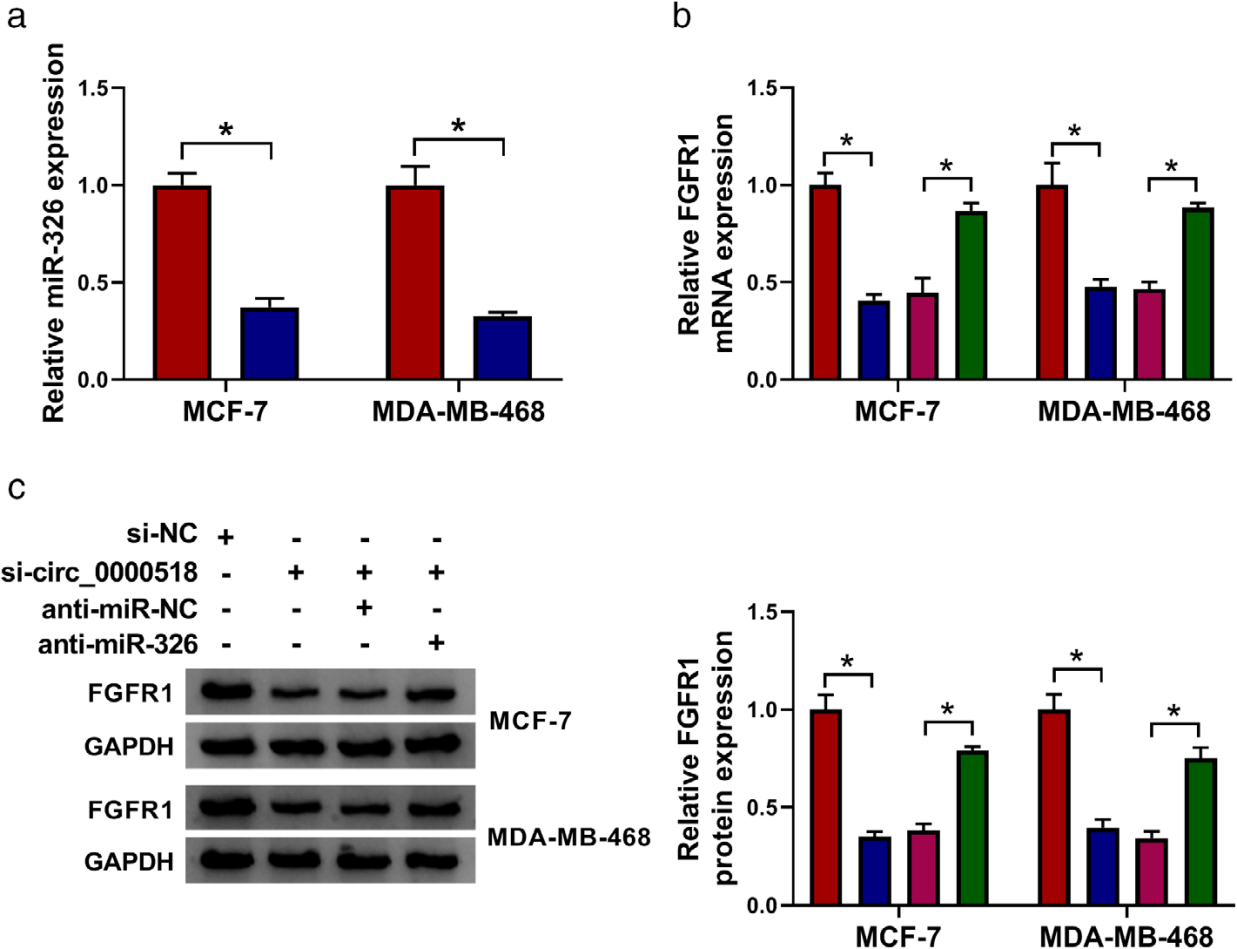
Circ_0000518 regulated FGFR1 expression through sponging miR‐326 in BC cells. (**a**) QRT‐PCR was carried out to assess the levels of miR‐326 in MCF‐7 and MDA‐MB‐468 cells after anti‐miR‐NC or anti‐miR‐326 transfection. (**b** and **c**) The levels of FGFR1 mRNA and protein in MCF‐7 and MDA‐MB‐468 cells transfected with si‐NC, si‐circ_0000518, si‐circ_0000518+anti‐miR‐NC, or si‐circ_0000518+anti‐miR‐326 were examined via qRT‐PCR or western blotting. The experiments were repeated 3 times. Data are shown as mean ± standard deviation. *P < 0.05. (**a**) (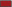) Anti‐miR‐NC and (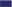) anti‐miR‐326. (**b**) (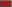) si‐NC, (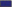) si‐circ_0000518, (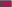) si‐circ_0000518 + anti‐miR‐NC, and (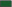) si‐circ_0000518 + anti‐miR‐326. (**c**) (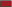) si‐NC, (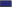) si‐circ_0000518, (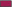) si‐circ_0000518 + anti‐miR‐NC, and (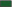) si‐circ_0000518 + anti‐miR‐326.

Based on the above findings, we further investigated whether circ_0000518 acted as a competing endogenous RNA (ceRNA) in BC cells. The results showed that miR‐326 expression was markedly reduced in MCF‐7 and MDA‐MB‐468 cells after transfection with anti‐miR‐326 compared to the control anti‐miR‐NC (Fig 4a). Furthermore, circ_0000518 silencing downregulated the levels of FGFR1 mRNA and protein in MCF‐7 and MDA‐MB‐468 cells, but this trend was recovered after anti‐miR‐326 transfection (Fig 4b,c). These data indicated that circ_0000518 regulated FGFR1 expression through sponging miR‐326 in BC cells.

### MiR‐326 inhibitor abolished circ_0000518 silencing‐mediated influence on the malignancy of BC cells

**Figure 5:**
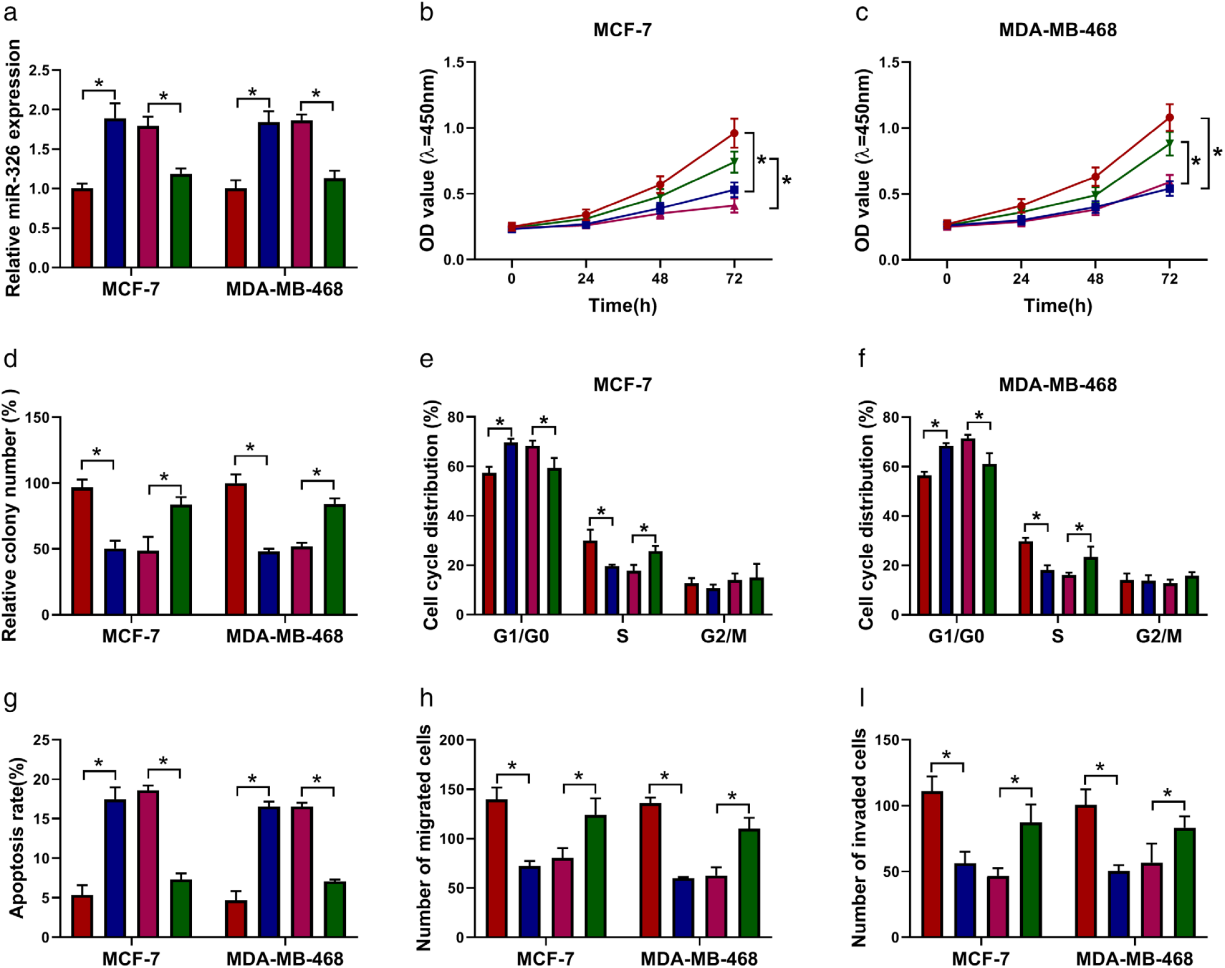
Circ_0000518 regulated BC progression via sponging miR‐326. (**a**–**i**) MCF‐7 and MDA‐MB‐468 cells were transfected with si‐NC, si‐circ_0000518, si‐circ_0000518 + anti‐miR‐NC, or si‐circ_0000518 + anti‐miR‐326. (**a**) Expression levels of miR‐326 in MCF‐7 and MDA‐MB‐468 cells were detected through qRT‐PCR. (**b**–**i**) The proliferation, colony formation, cell cycle progression, apoptosis, migration, and invasion of MCF‐7 and MDA‐MB‐468 cells were evaluated through CCK‐8, colony formation, flow cytometry, or transwell assays. The experiments were repeated three times. Data are shown as mean ± standard deviation. *P < 0.05. (**a**) (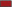) si‐NC, (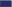) si‐circ_0000518, (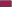) si‐circ_0000518 + anti‐miR‐NC, and (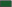) si‐circ_0000518 + anti‐miR‐326. (**b**) MCF‐7 (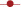) si‐NC, (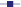) si‐circ_0000518, (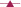) si‐circ_0000518 + anti‐miR‐NC, and (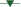) si‐circ_0000518 + anti‐miR‐326. (**c**) MDA‐MB‐468 (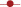) si‐NC, (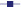) si‐circ_0000518, (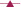) si‐circ_0000518 + anti‐miR‐NC, and (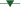) si‐circ_0000518 + anti‐miR‐326. (**d**) (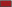) si‐NC, (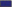) si‐circ_0000518, (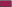) si‐circ_0000518 + anti‐miR‐NC, and (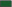) si‐circ_0000518 + anti‐miR‐326. (**e**) MCF‐7 (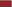) si‐NC, (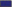) si‐circ_0000518, (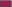) si‐circ_0000518 + anti‐miR‐NC, and (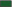) si‐circ_0000518+anti‐miR‐326. (**f**) MDA‐MB‐468 (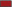) si‐NC, (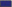) si‐circ_0000518, (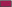) si‐circ_0000518 + anti‐miR‐NC, and (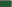) si‐circ_0000518 + anti‐miR‐326. (**g**) (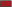) si‐NC, (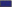) si‐circ_0000518, (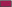) si‐circ_0000518 + anti‐miR‐NC, and (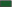) si‐circ_0000518 + anti‐miR‐326. (**h**) (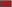) si‐NC, (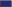) si‐circ_0000518, (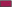) si‐circ_0000518 + anti‐miR‐NC, and (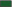) si‐circ_0000518 + anti‐miR‐326. (**i**) (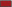) si‐NC, (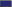) si‐circ_0000518, (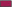) si‐circ_0000518 + anti‐miR‐NC, and (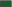) si‐circ_0000518 + anti‐miR‐326.

Given that circ_0000518 acted as a sponge for miR‐326, we further surveyed the mechanism between circ_0000518 and miR‐326 in BC progression. The results exhibited that miR‐326 expression was increased in circ_0000518‐silenced MCF‐7 and MDA‐MB‐468 cells, while this trend was reversed after miR‐326 inhibitor transfection (Fig 5a). Furthermore, the inhibitory influence of circ_0000518 knockdown on proliferation, colony formation, and cell cycle progression of MCF‐7 and MDA‐MB‐468 cells was overturned by miR‐326 inhibition (Fig 5b–f). Moreover, the elevation of the apoptotic rate of MCF‐7 and MDA‐MB‐468 cells caused by circ_0000518 inhibition was recovered after miR‐326 silencing (Fig 5g). Also, decreased miR‐326 expression overturned the repression of migration and invasion of MCF‐7 and MDA‐MB‐468 cells mediated by circ_0000518 inhibition (Fig 5h,i). Taken together, these data indicated that circ_0000518 modulated BC progression via sponging miR‐326.

### FGFR1 overexpression recovered miR‐326 mimic‐mediated impacts on the malignancy of BC cells

**Figure 6:**
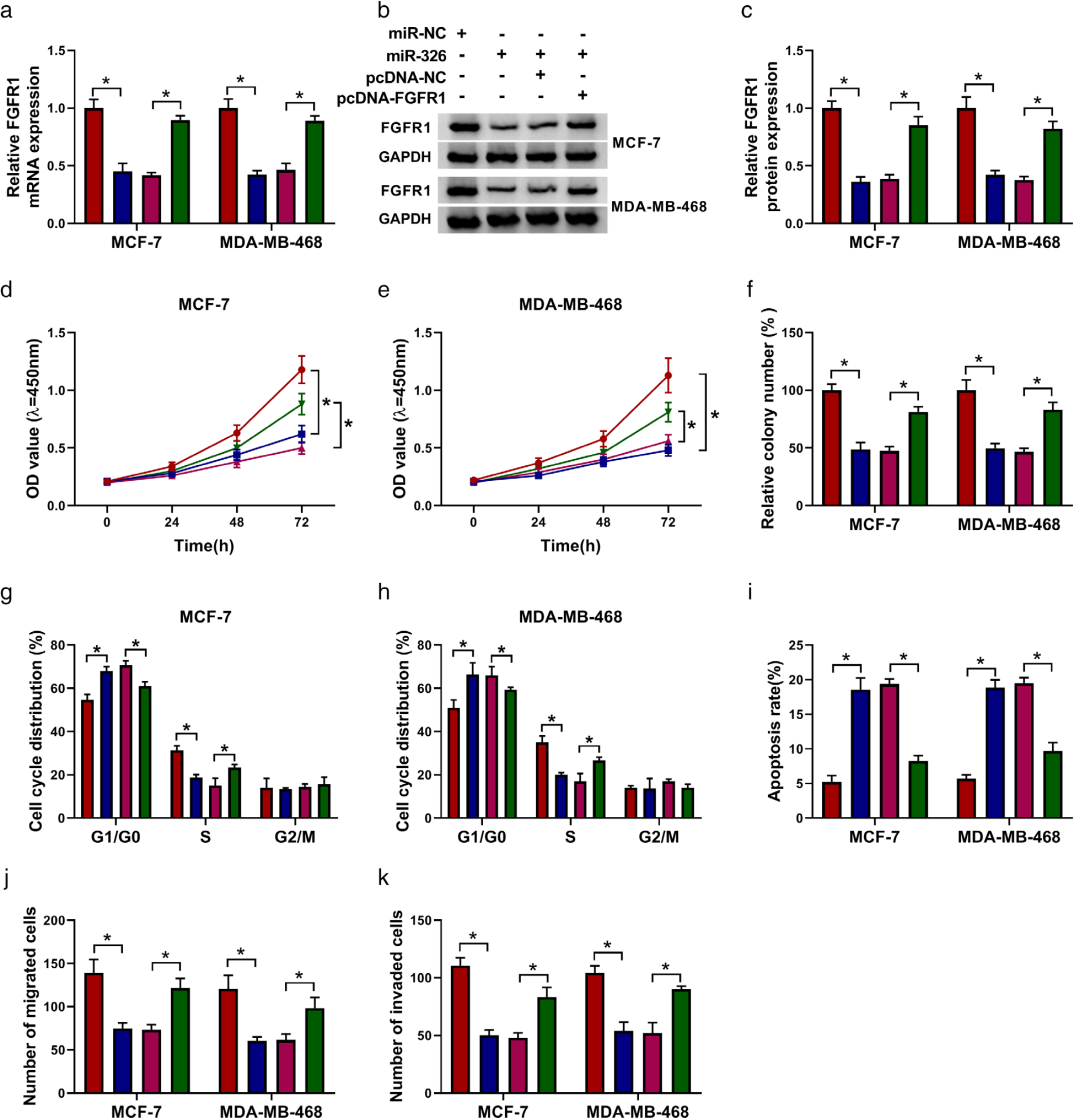
MiR‐326 regulated BC advancement via targeting FGFR1 in BC cells. (**a**–**k**) MCF‐7 and MDA‐MB‐468 cells were transfected with miR‐NC, miR‐326, miR‐326 + pcDNA‐NC, or miR‐326 + pcDNA‐FGFR1. (**a**–**c**) The mRNA and protein level of FGFR1 in MCF‐7 and MDA‐MB‐468 cells were detected with qRT‐PCR or western blotting. (**d**–**k**) The proliferation, colony formation, cell cycle progression, apoptosis, migration, and invasion of MCF‐7 and MDA‐MB‐468 cells were assessed through CCK‐8, colony formation, flow cytometry, or transwell assays. The experiments were repeated three times. Data are shown as mean ± standard deviation. *P < 0.05. (**a**) (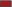) miR‐NC, (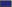) miR‐326, (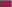) miR‐326+pcDNA‐NC, and (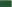) miR‐326 + pcDNA‐FGFR1. (**c**) (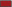) miR‐NC, (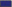) miR‐326, (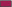) miR‐326 + pcDNA‐NC, and (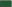) miR‐326 + pcDNA‐FGFR1. (**d**) MCF‐7 (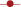) miR‐NC, (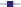) miR‐326, (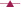) miR‐326 + pcDNA‐NC, and (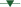) miR‐326 + pcDNA‐FGFR1. (**e**) MDA‐MB‐468 (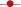) miR‐NC, (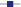) miR‐326, (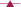) miR‐326 + pcDNA‐NC, and (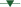) miR‐326 + pcDNA‐FGFR1. (**f**) (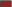) miR‐NC, (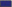) miR‐326, (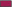) miR‐326 + pcDNA‐NC, and (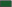) miR‐326 + pcDNA‐FGFR1. (**g**) MCF‐7 (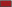) miR‐NC, (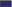) miR‐326, (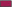) miR‐326 + pcDNA‐NC, and (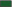) miR‐326 + pcDNA‐FGFR1. (**h**) MDA‐MB‐468 (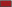) miR‐NC, (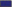) miR‐326, (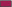) miR‐326 + pcDNA‐NC, and (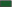) miR‐326 + pcDNA‐FGFR1. (**i**) (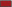) miR‐NC, (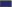) miR‐326, (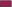) miR‐326 + pcDNA‐NC, and (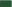) miR‐326 + pcDNA‐FGFR1. (**j**) (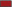) miR‐NC, (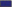) miR‐326, (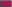) miR‐326 + pcDNA‐NC, and (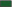) miR‐326 + pcDNA‐FGFR1. (**k**) (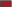) miR‐NC, (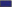) miR‐326, (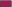) miR‐326 + pcDNA‐NC, and (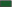) miR‐326 + pcDNA‐FGFR1.

Considering that FGFR1 was a downstream target for miR‐326 in BC, we explored whether miR‐326 played its role through FGFR1. We discovered that miR‐326 mimic inhibited the levels of FGFR1 mRNA and protein in MCF‐7 and MDA‐MB‐468 cells, while this influence was partly reversed after pcDNA‐FGFR1 transfection (Fig 6a–c). Furthermore, miR‐326 mimic cured cell proliferation, colony formation, and cell cycle progression in MCF‐7 and MDA‐MB‐468 cells, while this impact was overturned by FGFR1 overexpression (Fig 6d–h). Also, miR‐326 mimic elevated the apoptotic rate of MCF‐7 and MDA‐MB‐468 cells, but this tendency was overturned after FGFR1 upregulation (Fig 6i). Additionally, enhanced FGFR1 expression abolished the repressive impact of miR‐326 elevation on migration and invasion of MCF‐7 and MDA‐MB‐468 cells (Fig 6j,k). Collectively, these results indicated that miR‐326 played its role through targeting FGFR1 in BC cells.

### Circ_0000518 inhibition curbed tumor growth in vivo

**Figure 7:**
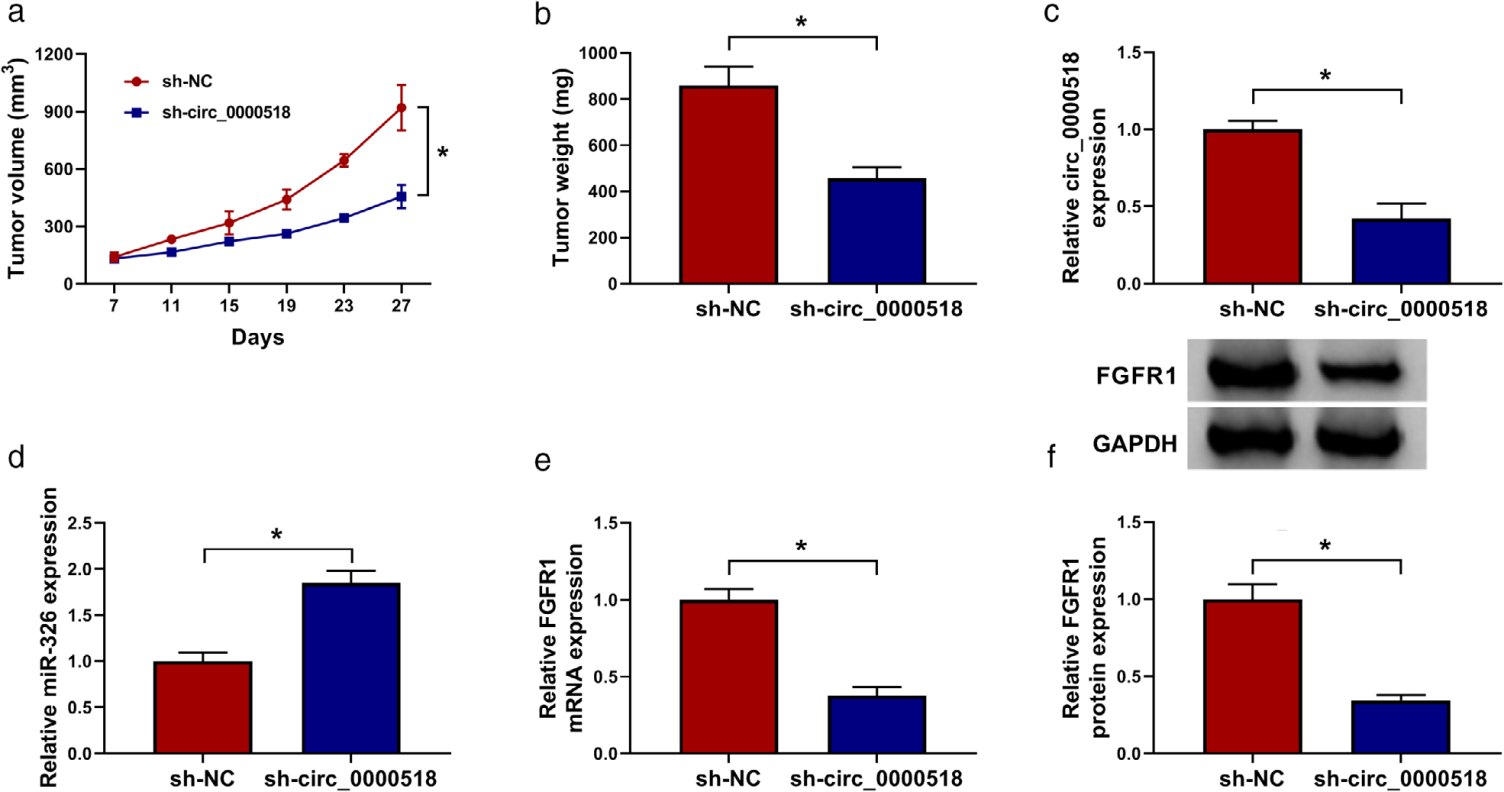
Circ_0000518 silencing could decrease BC growth in vivo. (**a**) The growth curves of tumor volume of the sh‐circ_0000518 and sh‐NC groups. (**b**) Xenograft tumor weight of the sh‐circ_0000518 and sh‐NC groups was assessed on day 27. (**c**–**f**) The levels of circ_0000518, miR‐326, FGFR1 mRNA and protein in mice tumor tissues of the sh‐circ_0000518 and sh‐NC groups were determined with qRT‐PCR or western blotting. The experiments were repeated three times. Data are shown as mean ± standard deviation. *P < 0.05. (**a**) (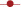) sh‐NC and (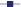) sh‐circ_0000518. (**b**) (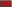) sh‐NC and (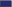) sh‐circ_0000518. (**c**) (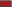) sh‐NC and (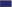) sh‐circ_0000518. (**d**) (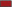) sh‐NC and (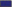) sh‐circ_0000518. (**e**) (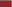) sh‐NC and (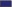) sh‐circ_0000518. (**f**) (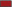) sh‐NC and (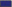) sh‐circ_0000518.

Knowing that circ_0000518 played a promoting role in BC cells in vitro, we further confirmed the role of circ_0000518 in BC in vivo through xenograft assay. We observed that tumor volume and weight were repressed and decreased in the sh‐circ_0000518 group relative to the sh‐NC group (Fig 7a,b). Also, circ_0000518 expression was decreased while miR‐326 expression was increased in mice tumor tissues of the sh‐circ_0000518 group in comparison to the sh‐NC group (Fig 7c,d). Moreover, the levels of FGFR1 mRNA and protein were decreased in the sh‐circ_0000518 group (Fig 7e,f). These data manifested that circ_0000518 silencing could decrease BC growth in vivo.

## Discussion

Emerging studies have demonstrated the vital roles of circRNAs in tumor development and progression.^20^ Some circRNAs have been verified as latent therapeutic targets and novel biomarkers in a series of diseases, including tumors.^21^ A previous report revealed that circRNA circ_KDM4C moderated doxorubicin resistance and impeded tumor growth in BC through increasing PBLD expression by sponging miR‐548p.^22^ Also, circRNA hsa_circ_0009362,^23^ circRNA circ_TFF1,^24^ and circRNA circ_circACAP2^25^ contributed to the advancement of BC. In the current study, circ_0000518 level was elevated in BC tissues and cells. Circ_0000518 silencing reduced tumor growth in vivo and facilitated cell cycle arrest, apoptosis, impeded proliferation, colony formation, migration, and invasion of BC cells in vitro. To the best of our knowledge, our research is the first to clarify the biological function of circ_0000518, and therefore we concluded that circ_0000518 exerted a carcinogenic role in BC.

Increasing evidence has demonstrated that circRNAs serve as ceRNAs taking part in the occurrence and progression of tumors. Recent research has shown that circRNA circ_SEPT9 facilitated triple‐negative BC carcinogenesis and advancement through activation of the LIF/STAT3 pathway via sponging miR‐637.^26^ Herein, we discovered that circ_0000518 served as a sponge for miR‐326 in BC cells. Previous studies have demonstrated that miR‐326 exerted a repressive role in diverse cancers.^27^, ^28^, ^29^, ^30^ Ghaemi *et al*. revealed that miR‐326 curbed BC progression via inactivation of the ErbB/PI3K pathway.^31^ Du *et al*. indicated that miR‐326 could impede invasion, migration, and proliferation of BC cells through targeting SOX12.^32^ Another study uncovered that miR‐326 expression was repressed by circRNA circ_TFF1, which contributed to BC development.^24^ Liang *et al*. claimed that miR‐326 mimic could elevate sensitivity of BC cells to doxorubicin through regulating MRP1 expression.^33^ In the research, miR‐326 expression was decreased in BC tissues and cells. Also, miR‐326 inhibitor reserved circ_0000518 silencing‐mediated impacts on proliferation, colony formation, cell cycle progression, apoptosis, migration, and invasion of BC cells. Therefore, these data indicated that circ_0000518 regulated BC progression via sponging miR‐326.

FGFR1 has been demonstrated as an oncogene in a series of cancers.^17^, ^18^ C11, an inhibitor of FGFR1, could curb angiogenesis and metastasis of BC.^34^ Previous research revealed that FGFR1 promoted resistance to endocrine therapy and acted as a possible target for BC.^35^ One report uncovered that FGFR1 expression was inhibited by miR‐361‐5p, which impeded BC cell invasion, proliferation, and glycolysis.^36^ Golfmann *et al*. unraveled that targeting both FGFR1 and VEGFR1 had a synergistic therapeutic effect in FGFR1/VEGFR1‐positive BC patients.^37^ In the present research, we revealed that FGFR1 was a target for miR‐326 in BC. Moreover, circ_0000518 regulated FGFR1 expression via sponging miR‐326. FGFR1 overexpression abolished the impact of miR‐326 mimic on proliferation, colony formation, cell cycle progression, apoptosis, migration, and invasion of BC cells. Therefore, we concluded that circ_0000518 regulated BC progression via modulating the miR‐326/FGFR1 axis.

In conclusion, our research showed the critical role of circ_0000518 in BC. Circ_0000518 accelerated BC progression via elevating FGFR1 expression by sponging miR‐326. The findings highlight the prospect of circ_0000518 as a new target for BC treatment.

## Disclosure

The authors declare that there are no competing interests associated with the manuscript.

## Supporting information


**Figure S1** Expression of five circRNAs in BC tissues. QRT‐PCR revealed the expression of hsa_circRNA_001846, hsa_circRNA_000518, hsa_circRNA_002172, hsa_circRNA_002144, and hsa_circRNA_000166 in BC tissues (10 random samples) and paired para‐carcinoma tissues. The experiments were repeated three times. Data were exhibited as mean ± standard deviation. *P < 0.05.

**Figure S2** Influence of circ_0000518 overexpression on the malignant behaviors of BC cells. (**A**) QRT‐PCR revealed the overexpression efficiency of circ_0000518 in MCF‐7 and MDA‐MB‐468 cells. (**B**–**I**) Effects of circ_0000518 overexpression on proliferation, colony formation, cell cycle progression, apoptosis, migration, and invasion of MCF‐7 and MDA‐MB‐468 cells were determined using CCK‐8, colony formation, flow cytometry, or transwell assays. The experiments were repeated three times. Data were exhibited as mean ± standard deviation. *P < 0.05.
