# Supplementary figures and images for: Hsa_circRNA_0000518 facilitates breast cancer development via regulation of the miR‐326/FGFR1 axis
Source: Thorac Cancer. 2020 Oct 1;11(11):3181–92. doi: 10.1111/1759-7714.13641 (PMC7606003; doi:10.1111/1759-7714.13641)

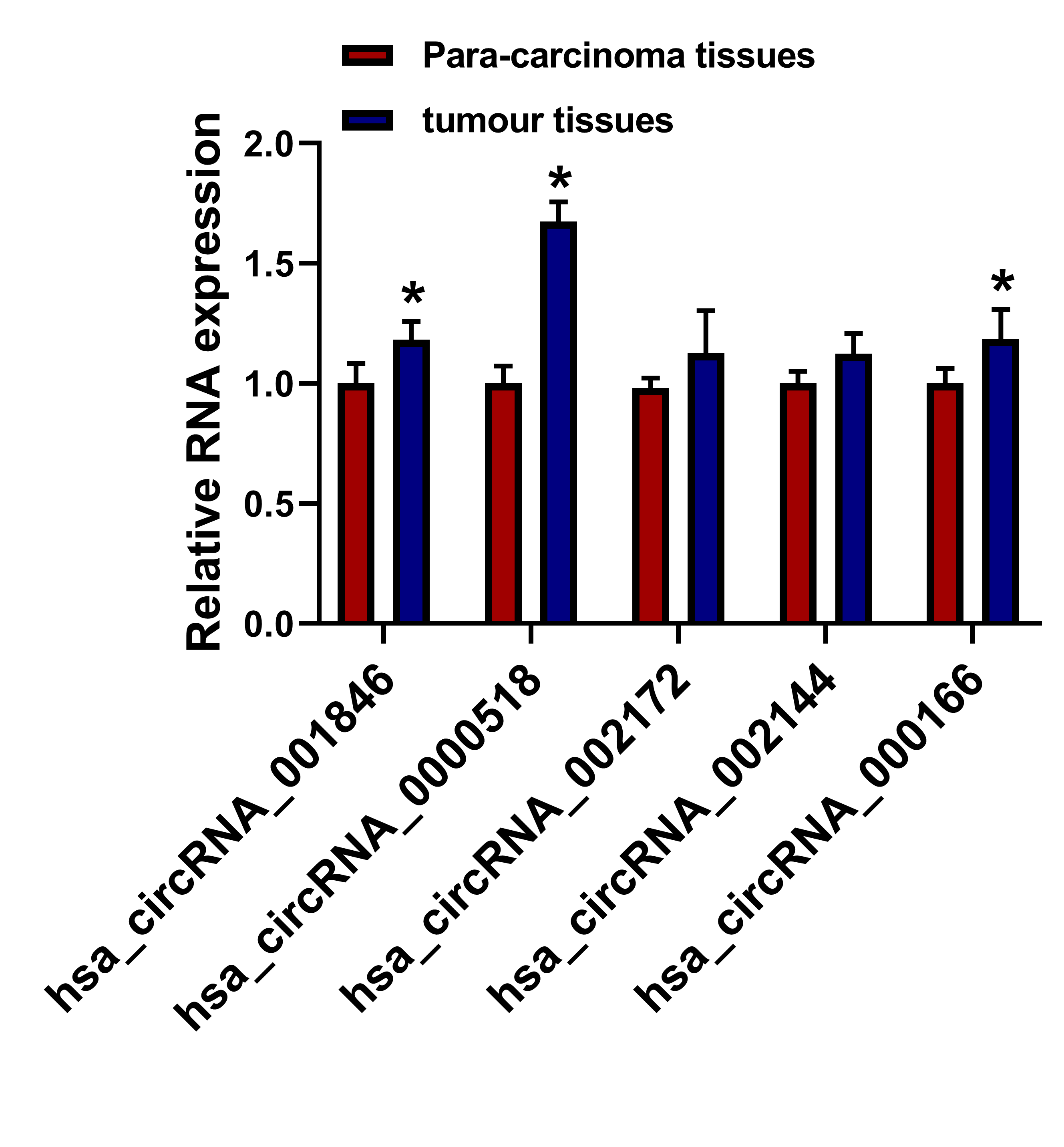

Supplement: Supplementary file 1 — Figure S1 Expression of five circRNAs in BC tissues. QRT‐PCR revealed the expression of hsa_circRNA_001846, hsa_circRNA_000518, hsa_circRNA_002172, hsa_circRNA_002144, and hsa_circRNA_000166 in BC tissues (10 random samples) and paired para‐carcinoma tissues. The experiments were repeated three times. Data were exhibited as mean ± standard deviation. *P < 0.05. [file TCA-11-3181-s001.tif]

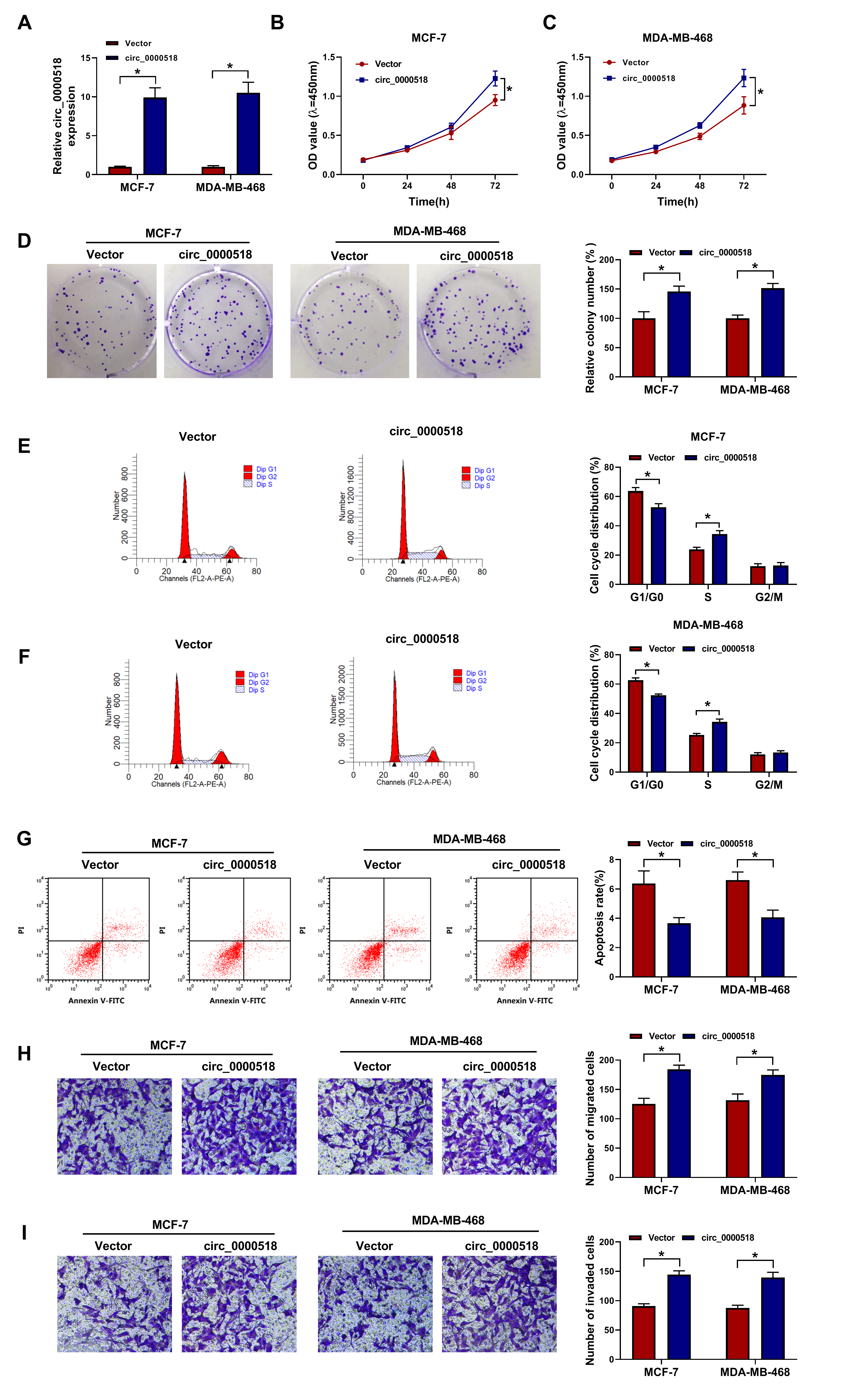

Supplement: Supplementary file 2 — Figure S2 Influence of circ_0000518 overexpression on the malignant behaviors of BC cells. (A) QRT‐PCR revealed the overexpression efficiency of circ_0000518 in MCF‐7 and MDA‐MB‐468 cells. (B–I) Effects of circ_0000518 overexpression on proliferation, colony formation, cell cycle progression, apoptosis, migration, and invasion of MCF‐7 and MDA‐MB‐468 cells were determined using CCK‐8, colony formation, flow cytometry, or transwell assays. The experiments were repeated three times. Data were exhibited as mean ± standard deviation. *P < 0.05. [file TCA-11-3181-s002.tif]
